# miR-155 Deletion in Female Mice Prevents Diet-Induced Obesity

**DOI:** 10.1038/srep22862

**Published:** 2016-03-08

**Authors:** Andrew D. Gaudet, Laura K. Fonken, Liubov V. Gushchina, Taryn G. Aubrecht, Santosh K. Maurya, Muthu Periasamy, Randy J. Nelson, Phillip G. Popovich

**Affiliations:** 1 Department of Neuroscience, Wexner Medical Center, The Ohio State University, Biomedical Research Tower 6^th^ floor 460 W. 12^th^ Ave Columbus, OH 43210 USA; 2 Center for Brain and Spinal Cord Repair, Wexner Medical Center, The Ohio State University, Biomedical Research Tower 6^th^ floor 460 W. 12^th^ Ave Columbus, OH 43210 USA; 3 Institute for Behavioral Medicine Research, Wexner Medical Center, The Ohio State University, Biomedical Research Tower 6^th^ floor 460 W. 12^th^ Ave Columbus, OH 43210 USA; 4 Department of Physiology and Cell Biology, Wexner Medical Center, The Ohio State University, Biomedical Research Tower 6^th^ floor, 460 W. 12^th^ Ave, Columbus, OH 43210 USA

## Abstract

Obesity is a growing epidemic in developed countries. Obese individuals are susceptible to comorbidities, including cardiovascular disease and metabolic disorder. Increasing the ability of adipose tissue to expend excess energy could improve protection from obesity. One promising target is microRNA (miR)-155-5p. We demonstrate that deletion of miR-155 (-5p and -3p) in female mice prevents diet-induced obesity. Body weight gain did not differ between wild-type (WT) and miR-155 knockout (KO) mice fed control diet (CD); however, miR-155 KO mice fed high-fat diet (HFD) gained 56% less body weight and 74% less gonadal white adipose tissue (WAT) than WT mice. Enhanced WAT thermogenic potential, brown adipose tissue differentiation, and/or insulin sensitivity might underlie this obesity resistance. Indeed, miR-155 KO mice on HFD had 21% higher heat release than WT HFD mice. Compared to WT adipocytes, miR-155 KO adipocytes upregulated brown (Ucp1, Cidea, Pparg) and white (Fabp4, Pnpla2, AdipoQ, Fasn) adipogenic genes, and glucose metabolism genes (Glut4, Irs1). miR-155 deletion abrogated HFD-induced adipocyte hypertrophy and WAT inflammation. Therefore, miR-155 deletion increases adipogenic, insulin sensitivity, and energy uncoupling machinery, while limiting inflammation in WAT, which together could restrict HFD-induced fat accumulation. Our results identify miR-155 as a novel candidate target for improving obesity resistance.

Obesity is a growing epidemic in Western countries with serious public health and financial implications^1^,^2^. Obesity occurs when energy expenditure is less than caloric intake over time^3^. Lifestyle interventions that improve energy balance (e.g., altering exercise or diet regimens) can be ineffective due to non-compliance and compensatory mechanisms^4^,^5^; therefore, discovering new therapeutic targets may improve protection from obesity.

microRNAs (miRs) are small non-coding RNAs that can modulate transcriptional networks, influencing biological processes throughout the body. miRs regulate gene expression by degrading complementary mRNA targets^6^; individual miRs can target hundreds of mRNAs simultaneously (an average miR targets ~400 mRNAs^7^). By manipulating specific miRs, it may be possible to control intracellular signaling pathways that predispose for excess fat accumulation^8^.

miR-155-5p is one promising candidate for protecting against obesity. miR-155-5p may regulate the development and maintenance of obesity via several signaling pathways, including browning, adipogenic, and inflammatory programs (Supplemental Table S1). First, miR-155-5p has been implicated in biasing adipocyte differentiation towards a white, rather than brown/beige phenotype. Brown adipocytes are a key site of energy expenditure; shifting adipocyte differentiation towards a brown adipocyte-like phenotype may increase energetic efficiency in mammals^9^,^10^,^11^,^12^,^13^. Second, miR-155-5p targets RNAs that control lipolysis^14^,^15^ (e.g., via Pnpla2^16^,^17^), which could affect energy storage in adipocytes. Finally, miR-155-5p may influence adipose tissue accumulation by regulating inflammatory pathways. Obesity triggers chronic low-grade inflammation, which in turn enhances fat accumulation and pathology^18^,^19^. Importantly, miR-155 activates pro-inflammatory pathways^20^. Thus, these diverse effects of miR-155-5p may act together to exacerbate obesity. This led us to hypothesize that deletion of miR-155 (both -5p and -3p) would confer resistance to obesity.

Here, we report that female miR-155 KO mice are protected from HFD-induced obesity and WAT accumulation. Wildtype (WT) and miR-155 knockout (KO) mice were placed on control diet (CD) or HFD for 12 weeks. Compared to WT mice, miR-155 KO mice on HFD showed reduced body and WAT weight gain, improved glucose tolerance and enhanced heat release. Furthermore, obesity resistance in KO mice is associated with reduced inflammatory signaling in WAT, enhanced adipogenic differentiation and increased adipocyte expression of brown adipose-related genes. Our data indicate that protection from obesity may be achieved by limiting the regulatory effects of miR-155.

## Results

### miR-155 deletion protects against HFD-induced obesity

**Figure 1:**
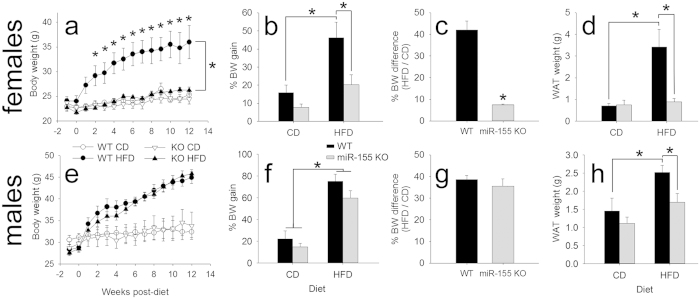
miR-155 deletion prevents HFD-induced obesity in female mice, and WAT accumulation in females and males. (**a**) Female miR-155 KO mice are protected against HFD-induced obesity. The body weight (BW) of female miR-155 KO mice on HFD was not significantly different from KO mice on CD, and was 56% lower than WT mice on HFD. (**b**) Percent BW gain from start of experiment in female WT and miR-155 KO mice on CD or HFD. (**c**) Percent BW difference between HFD/CD animals shows that HFD caused 35% less weight gain in KO mice. (**d**) Compared to WT mice on HFD, female miR-155 KO mice on HFD had lower final WAT mass. (**e–g**) For males, both WT and miR-155 KO mice on HFD gained significantly more BW than respective CD controls. (**h**) Compared to WT mice on HFD, miR-155 KO male WAT weight was reduced. * indicates p < 0.05.

We hypothesized that miR-155 KO mice would be protected against diet-induced obesity. HFD increased body weight 2.9-fold in WT mice (as compared with WT mice fed CD). Conversely, HFD-induced body weight gain was abolished in miR-155 KO female mice: miR-155 KO mice fed HFD gained no more than KO mice fed standard chow and 56% less than WT mice on HFD (p < 0.05 from weeks 2–12, inclusive) (Fig. 1a–c).

Changes in body weight corresponded with accumulation of gonadal WAT depot weight (Fig. 1d). WAT from WT mice on HFD weighed 285% more than WAT from miR-155 KO HFD mice. WAT weight from female miR-155 KO mice fed HFD was not significantly different from WT or KO mice fed control chow. Proportional weight of the gonadal fat pad is a reliable estimate of body fat composition in both normal and obese mice^21^; gonadal fat weight strongly correlates with percent body fat in both males and females (r = 0.97). These body and WAT weight differences corroborate data from a pilot experiment (using WT and miR-155 KO mice from non-littermates; Supplemental Fig. S1a–d) and indicate that miR-155 deletion protects female mice from HFD-induced weight gain and WAT accumulation (Fig. 1).

Whether male miR-155 KO mice were similarly protected from the effects of diet-induced obesity was evaluated in follow-up experiments. Although WT and miR-155 KO males fed HFD did not have overt differences in body weight (Fig. 1e–g), miR-155 deletion reduced WAT weight gain by 50% (Fig. 1h). Therefore, miR-155 deletion prevented HFD-induced accumulation of WAT in both females and males. Normal developmental patterns of body weight gain occurred in both female and male miR-155 KO mice (Supplemental Fig. 1e,f).

**Figure 2:**
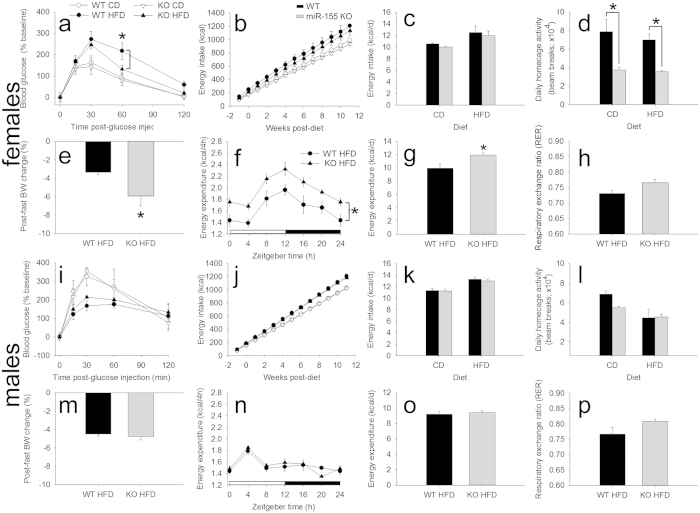
Female, but not male miR-155 KO mice display altered glucose metabolism, energetics, and metabolism. (**a**) Glucose metabolism in female WT and miR-155 KO mice fed HFD. At 60 min post-injection, miR-155 KO mice reduced blood glucose by 39% compared to WT HFD mice. (**b,c**) Regardless of diet, energy intake was not altered in female miR-155 KO mice compared to WT mice. (**d**) Female miR-155 KO mice were ~50% less active in their home cages on either diet. (**e**) After overnight fasting, female miR-155 KO mice on HFD lost 78% more body mass than did WT mice. (**f,g**) Compared to WT mice, female miR-155 KO mice on HFD increased energy expenditure (heat production) by 14%. (**h**) Respiratory exchange ratio (RER) was not altered in female miR-155 KO mice. (**i–p**) The same parameters were measured in male WT and miR-155 KO mice. In males, none of these metabolic indicators were altered between genotypes. * indicates p < 0.05.

Obesity is associated with reduced ability to metabolize glucose and increased risk of diabetes. Therefore, glucose metabolism after an overnight fast was examined using an intraperitoneal glucose tolerance test. In female miR-155 KO mice, glucose levels recovered more quickly than in WT mice (Fig. 2a; Supplemental Fig. S2a), indicating that miR-155 deletion improves glucose metabolism in female mice fed HFD. Glucose metabolism was not significantly altered in male mice fed HFD (Fig. 2i, Supplemental Fig. S3a).

### miR-155 deletion increases energy expenditure

To further understand mechanisms underlying the anti-obesity effects of miR-155 deletion, female and male mice were used to study energy balance and metabolism (Fig. 2; Supplemental Figs S2 and S3). The data indicate that regardless of diet, cumulative and daily energy intake did not differ between female WT and miR-155 KO mice (Fig. 2b,c). Thus, the anti-obesity effects of miR-155 deletion are not related to genotype-specific differences in energy intake. Moreover, it does not appear that miR-155 deletion protects against weight gain by increasing activity. Indeed, miR-155 KO mice gained less body weight on HFD, yet were ~50% less active in their home cages than WT mice (Fig. 2d).

miR-155 deletion could protect against obesity in female mice by enhancing metabolic rate (Fig. 2e–h (HFD mice only), Supplemental Fig. S2 (CD and HFD mice)). Compared to female WT mice, miR-155 KO mice lost 78% more body weight after overnight fast (Fig. 2e). Furthermore, female miR-155 KO mice increased energy expenditure (heat production) by 14% over WT mice, supporting a role for miR-155 deletion in increasing metabolic rate (Fig. 2f,g). However, female WT and miR-155 KO mice had comparable rectal temperatures throughout the day (Supplemental Fig. S2g). Respiratory exchange ratio (RER) did not differ between the genotypes (Fig. 2h), suggesting that miR-155 deletion does not affect resting fat or carbohydrate metabolism^22^. Interestingly, male mice did not show any significant differences in these parameters (Fig. 2i–p; Supplemental Fig. S3); thus, the metabolic effects of miR-155 deletion are more prominent in female mice.

### In WAT, miR-155 KO adipocytes are resistant to HFD-induced hypertrophy and inflammation

**Figure 3:**
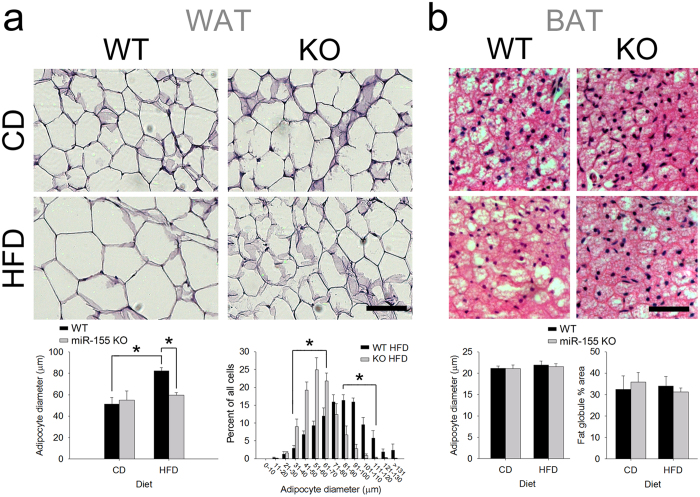
miR-155 deletion in females abrogated HFD-induced WAT adipocyte hypertrophy. (**a**) Representative images show hematoxylin-eosin-stained WAT from WT and miR-155 KO female mice fed either CD or HFD. In WT mice, HFD increased average adipocyte cross-sectional diameter by 61%. In miR-155 KO mice, the effect of HFD on adipocyte size was abolished. The histogram that displays different adipocyte diameters demonstrates reduced KO adipocyte size. Scale bar = 100 μm. (**b**) In intrascapular BAT, brown adipocyte diameter and morphology was not significantly altered by miR-155 deletion or by HFD. Hematoxylin and eosin were used to visualize BAT cytoarchitecture. Data were analyzed using overall average diameter and by measuring percent area of fat globules. Scale bar = 50 μm. * indicates p < 0.05.

To establish whether miR-155 deletion affects WAT cell phenotype^23^,^24^, we measured adipocyte size and mRNA expression in WAT of female WT and miR-155 KO mice. As expected, HFD increased the size of WT adipocytes (61% larger vs. WT adipocytes from CD mice). However, miR-155 deletion prevented HFD-induced increase in adipocyte size (Fig. 3a). Adipocytes from miR-155 KO mice fed HFD were 38% smaller than adipocytes from WT mice fed HFD. There were no significant differences in brown adipocyte diameter or morphology (Fig. 3b).

**Figure 4:**
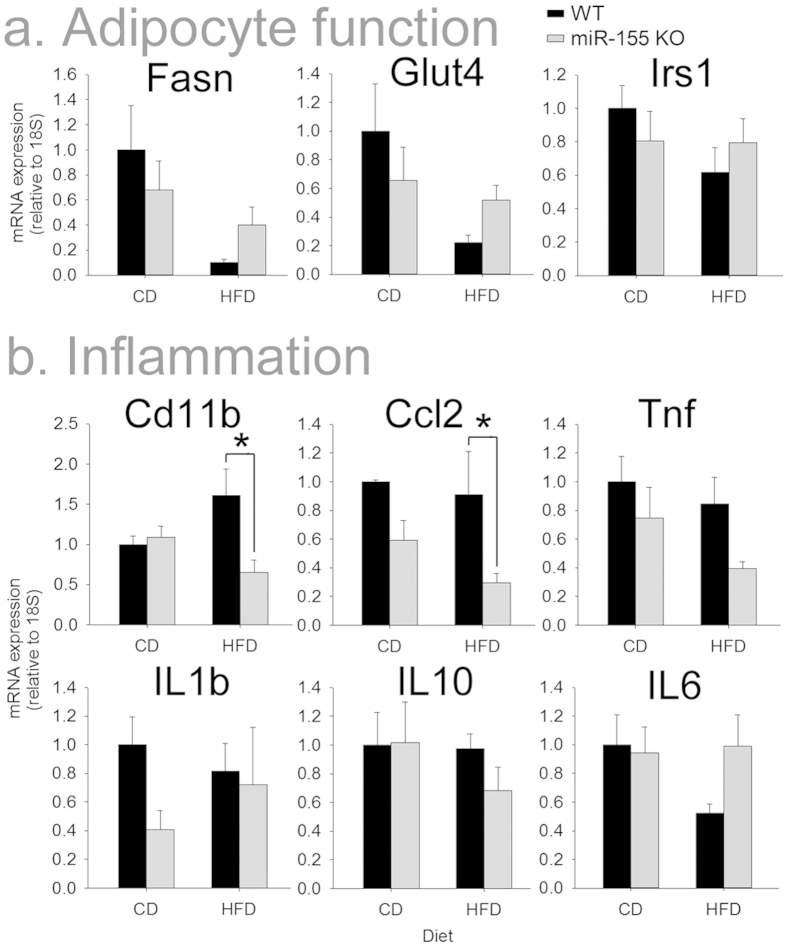
Female miR-155 KO mice on HFD did not have altered adipogenic or insulin sensitivity genes, but had reduced expression of inflammatory mRNAs in WAT. (**a**) Expression of the lipogenic gene Fasn and the insulin sensitivity genes Glut4 and Irs1 were not significantly altered in WAT from KO mice. (**b**) Compared to WAT from WT mice on HFD, miR-155 KO WAT had reduced Cd11b and Ccl2, suggesting that miR-155 deletion dampened obesity-related inflammatory responses. * indicates p < 0.05.

The extent and phenotype of adipocyte differentiation can affect susceptibility to obesity. Several adipogenesis regulators are targeted by miR-155^14^,^25^, suggesting the microRNA could regulate adipocyte differentiation. Thus, expression of genes involved in adipogenesis and insulin resistance was examined in WAT from female WT and KO mice. Expression of genes encoding fatty acid synthase (*Fasn*), the glucose transporter *Glut4* and insulin receptor substrate 1 (*Irs1*) was not altered in WAT from KO mice (Fig. 4a).

Diet-induced obesity causes inflammatory macrophages to infiltrate fat, which in turn enhance fat accumulation and pathology^19^. Macrophages with inhibited/deleted miR-155 have substantially diminished capacity for inflammatory signaling^20^,^26^. Therefore, we examined whether miR-155 deletion affected inflammatory gene expression in WAT (Fig. 4b). WAT *Cd11b* (a marker for macrophages) and *Ccl2* (a monocyte chemotactic factor) mRNA expression was unaffected by miR-155 deletion in mice fed CD; however, expression of both genes was significantly reduced in miR-155 KO mice fed HFD. Expression of *Tnf, Il-1b, Il-10,* and *Il-6* mRNA in WAT were not significantly affected by miR-155 deletion.

### WT mice reconstituted with KO bone marrow display modest obesity resistance

**Figure 5:**
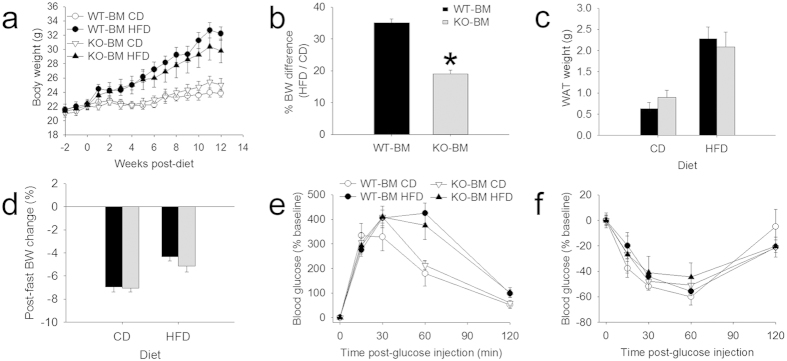
Transplant of miR-155 KO bone marrow (BM) into female WT host mice conferred limited obesity resistance. (**a,b**) Compared to control mice receiving WT bone marrow (WT-BM) transplant, chimeric mice that received miR-155 KO bone marrow (KO-BM) were partially protected from HFD-induced weight gain. (**b**) Percent body weight gain shows that HFD elicits 50% less weight gain in KO-BM mice. (**c**) WAT weight was not significantly altered in mice with miR-155 KO BM. (**d**) After overnight fast, mice with WT-BM or KO-BM lost similar percent body weight. (**e**) Glucose tolerance was not different between the two chimeras. (**f**) After insulin injection, chimeras with either WT or KO BM responded similarly. * indicates p < 0.05.

Reduced expression of *Cd11b* and *Ccl2* mRNA (Fig. 4) could indicate that deleting miR-155 reduces macrophage infiltration into WAT. This may represent a causal mechanism for how miR-155 deletion prevents weight gain in mice fed HFD. To test this hypothesis directly, female WT mice received whole-body irradiation then were reconstituted with female miR-155 KO bone marrow (miR-155 KO bone marrow → WT host mice; KO-BM). When these chimeric mice were fed HFD, their body weight was reduced 8% relative to that of control WT bone marrow → WT host mice (WT-BM; not significant; Fig. 5a). However, relative to their respective CD controls, KO-BM mice on HFD gained 50% less than WT-BM mice (Fig. 5b). KO-BM mice showed no significant differences in WAT weight, fasted percent body weight loss, glucose tolerance, or insulin tolerance (Fig. 5c–f). Therefore, although miR-155 KO macrophages confer some protection against obesity, it appears likely that miR-155 deletion mainly improves resistance to obesity through other mechanisms.

### Compared to WT cells, miR-155 KO white adipocytes increase adipogenic and BAT-related genes

**Figure 6:**
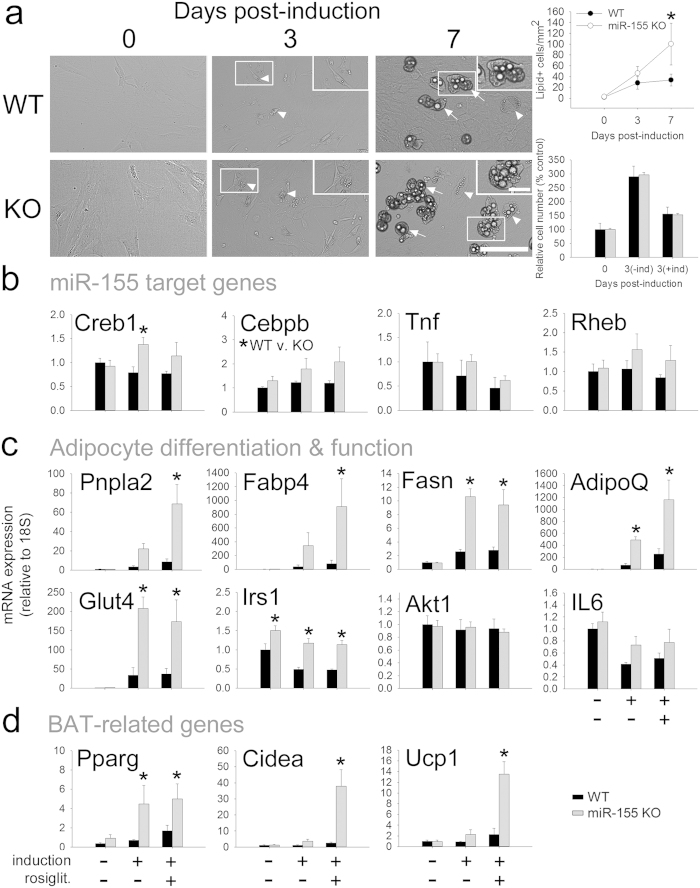
miR-155 KO preadipocytes from females differentiate more readily and express higher brown adipocyte mRNAs. (**a**) Live imaging of WT and KO WAT-derived preadipocytes at 0, 3, and 7 d post-induction. Arrowheads indicate small lipid-containing cells; arrows delineate large, more mature lipid-laden cells. At 7 d post-induction, miR-155 KO adipocyte cultures had 197% higher density of lipid-containing cells, but no significant difference in proliferation. (**b**) At 5d post-induction, expression of the miR-155 target mRNAs Creb1 and Cebpb were significantly higher in miR-155 KO adipocytes. Tnf (a miR-155-stabilized mRNA) and Rheb expression was not altered in miR-155 KO adipocytes. (**c**) miR-155 KO adipocytes expressed higher adipocyte differentiation and insulin sensitivity markers than WT adipocytes. (**d**) Compared to differentiated WT adipocytes, miR-155 KO adipocytes strongly increased expression of brown adipose tissue-related genes. * indicates p < 0.05. Scale bars = 100 μm (large images); 25 μm (insets).

As mentioned above, miR-155 could enable susceptibility to obesity by manipulating adipocyte factors and differentiation. *In vivo*, differentiation and browning of preadipocytes occurs asynchronously^27^ and is complicated by the presence of other cell types. Further, *in vivo* expression of these genes was only examined in WAT during chronic obesity/HFD. Thus, cultured white preadipocytes^28^ were used to systematically assess differentiation and browning potential of WT and KO cells. First, we explored whether miR-155 deletion influenced preadipocyte differentiation. Morphologically, mature adipocytes are distinguished by the presence of lipid droplets^28^. After 7 d differentiation, 197% more miR-155 KO adipocytes contained lipid droplets (Fig. 6a), suggesting that KO adipocytes differentiated more readily. miR-155 KO adipocytes did not show altered proliferative capacity at 3 d post-induction (without or with induction medium; Fig. 6a).

Obesity protection in miR-155 KO mice was likely due to altered adipocyte metabolism and/or enhanced energy release, so we hypothesized that miR-155 deletion would increase adipocyte expression of relevant miR-155 targets, of lipid metabolism (fatty acid breakdown) genes, and of genes involved in uncoupling of oxidative phosphorylation (“browning”). Two validated miR-155 targets were upregulated in KO adipocytes (Fig. 6b). *Creb1*, a known miR-155 target and transcription factor upstream of master adipogenic genes, was increased in differentiated KO compared to WT adipocytes (+37%). *Cebpb* is a validated miR-155 target and adipogenic transcription factor that biases adipocytes towards a brown-like phenotype^29^. *Cebpb* was upregulated in KO cells (ANOVA, p < 0.05). Two other miR-155 targets, *Tnf* and *Rheb*, were not significantly altered in KO adipocytes.

In parallel with enhanced lipid accumulation, genes involved in adipogenesis and insulin sensitivity were more strongly upregulated in miR-155 KO adipocytes exposed to insulin-containing induction cocktail (without or with the PPAR agonist rosiglitazone) (Fig. 6c). Compared to WT adipocytes, KO cells strongly increased terminal white adipose markers *Pnpla2* and *Fabp4* (increased 68× and 1300×, respectively; with rosiglitazone). Differentiated KO cells also upregulated *Fasn* (increased 9× and 8×, with induction and induction + rosiglitazone, respectively) and the anti-inflammatory adipokine adiponectin (*AdipoQ*) (increased 490× and 1160×, with induction and induction + rosiglitazone, respectively). Genes that promote insulin sensitivity were increased in differentiated KO adipocytes, including *Irs1* (increased 2.3× higher than WT rosiglitazone) and *Glut4* (increased 207× and 172×, with induction and induction + rosiglitazone, respectively). Expression of *Il6* and *Akt1* was not altered in KO adipocytes. Together, these changes likely contribute to obesity resistance; for example, reduced obesity is observed after overexpression of *Pnpla2*^30^ or adiponectin^31^, and *Fabp4* deletion exacerbates obesity^32^.

Finally, we examined BAT-related genes in WT and miR-155 KO adipocytes (Fig. 6d). *Pparg* – a master adipogenic regulator that shifts cells towards a brown phenotype – was more robustly upregulated with induction in KO cells (increased 13× and 14×, with induction and induction + rosiglitazone respectively, vs. WT cells). Induction +  rosiglitazone treatment of KO adipocytes also markedly increased the BAT markers *Ucp1* (increased 13× vs. WT) and *Cidea* (increased 38× vs. WT). Together, these data show that miR-155 KO preadipocytes differentiate more readily with enhanced upregulation of brown adipocyte markers.

## Discussion

We report here that miR-155 is important for HFD-induced body weight and WAT gain, particularly in female mice. miR-155 KO female mice were completely protected against diet induced obesity: miR-155 KO mice on HFD did not gain significantly more weight than mice fed a CD. Similarly, KO female mice fed HFD were protected against excess WAT accumulation and impairments in glucose tolerance. The effects of miR-155 deletion in males were more modest: although miR-155 KO males on HFD did not show reduced body weight gain, they did gain less WAT weight compared to WT male mice on HFD. The obesity resistance of miR-155 KO female mice was not due to altered energy intake or activity; rather, miR-155 KO mice on HFD increased energy release compared to WT mice. Changes in energy release may be mediated by adipocyte regulation as preadipocytes from miR-155 KO mice showed enhanced differentiation capacity and were biased towards a brown phenotype. This shift towards a brown-like cell likely enhances energy expenditure (over storage as fat). Together, our data suggest that miR-155 deletion improves obesity resistance, likely by modestly reducing WAT inflammation and by biasing preadipocytes towards a brown-like phenotype.

The efficacy of miR-155 deletion in females is remarkable; despite not affecting body weight during development or in adults maintained on standard chow, miR-155 deletion abolishes HFD-induced body weight gain in female mice. This contrasts with other obesity-resistant transgenic models that display only partial protection against diet-induced obesity. For example, PPAR-γ brain-specific KO mice on HFD weighed ~40% more than KO CD mice^33^; PPAR-γ adipose-specific KO mice weighed ~40% more than KO CD mice^34^; and CB1 cannabinoid receptor KO mice on HFD weighed ~30% more than KO mice on CD^35^. In contrast, data in this report show that miR-155 KO female mice did not weigh significantly more (7% higher) than KO mice on CD, highlighting the superior efficacy of miR-155 deletion.

Although miR-155 deletion reduced diet-induced WAT accumulation in both sexes, obesity resistance was particularly robust in female KO mice. Because women on Western diets are increasingly prone to obesity (e.g., 66% of female US adults were overweight/obese in 2012^1^), these data have high societal relevance. Although male rodents are considered to be the “gold standard” model for studying obesity^36^, our current data underscore the importance of considering sex-specific therapies or pathways that regulate diet-induced obesity^30^,^37^,^38^,^39^,^40^,^41^.

Differences in male and female miR-155 KO mice have been observed previously (in models of amyotrophic lateral sclerosis^42^ and lupus^43^). Furthermore, we previously reported behavioral differences in miR-155 male and female KO mice. Specifically, female, but not male, miR-155 KO mice show impairments in a rotarod task^44^, which may relate to the female specific reductions in locomotor activity^45^. Alternatively, sex differences in activity could be due to endocannabinoids^46^ or sex hormones^47^,^48^. Despite reduced home cage activity levels, female miR-155 KO mice had low adiposity. Why miR-155 KO mice were less active, yet still gained less weight and fat, is not clear. Overall, the results indicate that physiological benefits of miR-155 deletion (e.g., altered adipogenesis, adipocyte browning, fatty acid catabolism) were sufficient to overcome reduced activity and ultimately reduced adipose tissue accumulation.

miR-155 appears to control adipose tissue accumulation by affecting energy metabolism, as miR-155 KO mice on HFD release more energy as heat. In parallel, miR-155 KO adipocytes increase expression of genes involved in brown adipogenesis (*Cebpb, Creb, Pparg*), lipolysis (*Pnpla2* [a.k.a. *Atgl*]), and energy release (*Ucp1*), which could synergize to improve fat metabolism. C/EBP-β and CREB are transcription factors that can activate brown adipogenesis, partly by upregulating *Pparg*^29^,^49^,^50^,^51^. PPAR-γ is a crucial adipogenic transcription factor that drives brown cell differentiation^52^,^53^. The lipolysis factor PNPLA2 catalyzes triacylglycerol breakdown, thereby limiting adipocyte storage of excess energy (fat accumulation) and creating oxidized fatty acids^54^,^55^. These abundant free fatty acids fuel activity of uncoupling machinery (Ucp1) involved in cellular oxidative respiration in adipocytes, enhancing energy release^56^,^57^,^58^,^59^. In addition to expressing altered levels of adipogenic genes, miR-155 KO adipocytes robustly upregulated *Fabp4* [a.k.a. *Ap2*]), a gene involved in cell homeostasis. Obesity is exacerbated by removal of Fabp4^32^,^60^,^61^, a factor that likely helps maintain cell health/function during HFD challenge.

miR-155 deletion could also affect other obesity-related processes, including insulin sensitivity, availability of circulating hormones/proteins, and free fatty acid release (see Supplementary Table S1 for a partial list of relevant validated and predicted miR-155-5p targets). In humans, impaired IRS-1 signaling is associated with increased miR-155^62^. IRS-1 deletion reduces adipocyte differentiation^63^, and *in vivo* IRS-1 removal causes hyperinsulinemia and insulin resistance^64^,^65^. Insulin sensitivity is regulated by validated miR-155-5p targets, including TNF-α^66^,^67^ (which is stabilized by miR-155-5p) and Rheb^68^ (which is reduced by miR-155-5p). The satiety hormone leptin may also be regulated by miR-155: Adipocyte leptin expression is elevated by Rheb-mediated mTOR activation^69^. Thus, miR-155-5p may limit leptin expression via Rheb degradation. Another relevant circulating protein is adiponectin (AdipoQ), which was upregulated in cultured miR-155 KO adipocytes. Adiponectin enhances insulin sensitivity, drives fatty acid oxidation, and reduces macrophage inflammatory responses^70^,^71^; its transcription is increased by PPAR-γ^72^ (which was elevated in KO adipocytes) and reduced by TNF-α^73^,^74^. Differentiated KO adipocytes also increased Fasn, which enhances adipocyte differentiation^75^ and lipid accumulation^76^. Finally, KO adipocytes upregulated Glut4; increased Glut4 in mice on HFD could improve glucose tolerance and insulin resistance, and dampen adipocyte hypertrophy^77^,^78^,^79^. Therefore, miR-155-5p affects various pathways in parallel that may elicit adiposity and obesity.

Our data indicate that miR-155 deletion acts mainly through affecting adipocyte differentiation; however, originally we also hypothesized that miR-155 deletion may reduce obesity by dampening HFD-induced WAT inflammation. Adipose tissue acts as an endocrine organ^23^,^24^; when challenged by HFD, white adipocytes upregulate pro-inflammatory cytokines/chemokines that can enhance accumulation of macrophages^66^,^80^,^81^,^82^. In turn, these inflammatory macrophages propagate inflammation and WAT pathology, completing a harmful cycle that amplifies obesity and comorbidities^19^,^83^. In macrophages, miR-155-5p upregulation drives pro-inflammatory phenotype: miR-155-5p targets anti-inflammatory genes and stabilizes TNF-α mRNA^20^,^84^,^85^,^86^,^87^. Similarly, miR-155-5p in adipocytes could enhance cytokine production and related changes^14^. Our results show that myeloid cell-specific miR-155 deletion had limited effect on obesity protection in female mice. This may be due to the fact that miR-155-5p reduces expression of select inflammatory transcripts (despite its overall promotion of inflammatory bias in macrophages). For instance, the danger-associated molecular pattern protein HMGB1, which is implicated in obesity^88^,^89^,^90^, is a miR-155-5p target^91^.

miR-155 KO mice lack both the better-characterized miR-155-5p and the understudied miR-155-3p. miR-155 deletion was achieved by removing the majority of exon 2 of the miR-155-containing *bic* gene (970/1093 base pairs deleted)^92^. Since miR-155-5p is 88 base pairs from the start of *bic* exon 2 and miR-155-3p begins just 113 base pairs downstream, miR-155-3p must also have been deleted. Therefore, it is important to note that the effects of miR-155 deletion on obesity could be due to miR-155-5p and/or miR-155-3p deletion. Future studies could explore how miR-155-5p and -3p affect obesity and other pathologies.

## Conclusions

Because microRNAs target hundreds of mRNAs simultaneously, they represent a promising, yet under-explored, class of molecules for improving outcomes of pathology and disease. Our study demonstrates that selectively manipulating microRNAs could help alleviate obesity. miR-155-5p is an attractive target for combatting obesity: It has dual obesity-promoting effects via white adipogenesis and inflammation. Our results reveal that miR-155 deletion in mice robustly improves obesity resistance. miR-155 KO mice on HFD have increased energy release and reduced WAT inflammation and cell hypertrophy. miR-155 KO adipocytes express higher levels of uncoupling machinery, implying a brown adipocyte-like bias that could preferentially drive energy release (over storage). Therefore, we suggest that miR-155 inhibition is an attractive potential therapy for obesity.

## Methods

### Animals

All experimental procedures were approved by The Ohio State University Institutional Animal Care and Use Committee, and animals were maintained in accordance with the recommendations of the *National Institutes of Health and the Guide for the Care and Use of Laboratory Animals*. miR-155 heterozygous parents (kind gift from Amy Lovett-Racke; Jackson (Bar Harbor, ME, USA) stock 007745; C57BL/6 background)^92^ were bred to generate WT and miR-155 KO littermates that were used for all experiments. For studying developmental body weight gain, mice were weighed weekly from 2 to 13 weeks after birth. All animals were raised on standard irradiated chow (Harlan 7912 Teklad LM-485, 5.8% kcal%fat). For studying the effects of high-fat diet on body weight, mice aged 3–5 months were housed individually at 22 ± 2 °C under a 12:12 h light cycle (lights on at 7A EST). Food and water were provided *ad libitum*. Intake of regular chow was studied for two weeks (weeks −1 and 0) prior to adding special diets. Mice were then divided into control diet (CD; Research Diets D12450H, 10% kcal% fat) and high-fat diet (HFD; Research Diets D12451, 45% kcal% fat) groups (see Group Sizes below). For body weight and food weight (energy intake) were measured weekly for the remaining 13 weeks of the experiment. After 13 weeks on CD/HFD, mice were injected with an overdose of sodium pentobarbital and perfused intracardially with ice-cold PBS (0.1 M, pH 7.4).

To establish whether miR-155 deletion prevents obesity through dampened inflammation, chimeric mice with WT or miR-155 KO bone marrow (BM) were created (all WT recipient mice). Three-month old female WT C57BL/6 mice were γ-irradiated (5 Gy), immediately injected with freshly-isolated WT or miR-155 KO bone marrow cells via tail vein injection, and allowed to recover/reconstitute bone marrow for two months^93^. The mice were then fed CD or HFD.

### Group sizes for animal experiments

For the female WT-KO experiments (Figs 1 and 3, 4, 5, 6; littermates all from heterozygous parents): WT CD n = 4; WT HFD n = 5; KO CD n = 5; KO HFD n = 5. For the male WT-KO experiments (Fig. 1; littermates from heterozygous parents): WT CD n = 3; WT HFD n = 5; KO CD n = 4; KO HFD n = 4. For developmental body weight gain (Supplemental Fig. S1e,f): WT female n = 10; KO female n = 9; WT male n = 8; KO male n = 9. For the pilot female WT-KO experiments (Supplemental Fig. S1a–d; not littermates – WT or KO parents): WT HFD n = 5; KO HFD n = 5. For the female BM chimera experiments (Fig. 5; all WT hosts with female WT or KO bone marrow): WT-BM CD n = 6; WT-BM HFD n = 8; KO-BM CD n = 6; KO-BM HFD n = 8 (BM chimera mice were produced as previously described^93^).

### Locomotor activity analyses

Homecage locomotor activity was studied 10–11 weeks after dietary switch (CD/HFD start) using OPTO M3 animal activity monitors (Columbus Instruments)^94^. Data were continuously compiled using MDI software.

### Indirect calorimetry

At 10–11 weeks after dietary switch (CD/HFD start), animals were placed in individual metabolic chambers for 24 h (Comprehensive Lab Animal Monitoring System (CLAMS); Columbus Instruments, Columbus, OH, USA)^95^. The chambers are air-tight, other than two valves that permit air flow and measurement. Fresh air was delivered to the chamber and the composition of outgoing air was measured every 4 min by O_2_ and CO_2_ sensors. Respiratory Exchange Ratio (RER; [VCO_2_ released]/[VO_2_ consumed]) and energy expenditure (heat production) were automatically calculated. An RER of 1.0 represents oxidation of pure carbohydrates, whereas an RER of 0.7 represents oxidation of pure fatty acids. Data were combined into 24 h and 4 h bins for analyses. Energy expenditure data were adjusted using an analysis of covariance (ANCOVA) to nullify potential confounding effects of body weight differences between groups^96^.

### Glucose and insulin tolerance tests

Mice were fasted overnight (glucose tolerance) or for 4 h (insulin tolerance). For female and male WT/KO experiments, glucose tolerance was tested at 12 weeks post-CD/HFD; for the chimera experiment, glucose tolerance was tested at 11 weeks and insulin tolerance at 12 weeks post-CD/HFD. Body weight pre- and post-fast was recorded. The next morning, mice were injected with a bolus of glucose (1.5 mg/kg) or insulin (0.75 U/kg). Animals receiving both glucose and insulin tolerance tests had >7 d between tests to minimize stress. Tail blood was collected at 0, 30, 60, 90, and 120 min post-glucose injection. Blood glucose levels were immediately measured using a glucometer (Contour; Bayer, Mishawaka, IN, USA)^94^,^97^.

### Tissue processing and immunohistochemistry

Tissues were harvested and fixed in formalin for histological analyses. Gonadal WAT was collected and weighed immediately after dissection. Adipose tissue was placed in PBS, embedded in paraffin (Pathology Core Facility; Ohio State University Wexner Medical Center), then blocked and sectioned on a microtome at 100 μm. Hematoxylin and eosin stain was used to visualize WAT and BAT cytoarchitecture. Slides were dehydrated prior to coverslipping with permount mounting medium (Fisher Scientific, Waltham, MA, USA).

### Cell culture

Mouse primary preadipocytes were isolated and differentiated as previously described^28^,^98^. Inguinal fatpads from 4–6 week old female WT and miR-155 KO mice (n = 4 each) were isolated and mechanically/enzymatically digested (collagenase I; Life Technologies 17100, Carlsbad, CA, USA), then cultured in maintenance medium (DMEM/F12 (Life Technologies 11320) + 10% fetal bovine serum (FBS) (Life Technologies 26140) + 0.05% gentamicin (Life Technologies 15710)). Once the cells reached ~70% confluence, preadipocytes were differentiated with induction medium (DMEM/F12 + 5% FBS + 17 n*M* insulin (Sigma-Aldrich I0516, St. Louis, MO, USA) + 0.1 μM dexamethasone (Sigma-Aldrich D2915) + 250 μM 3-Isobutyl-1-methylxanthine (Sigma-Aldrich I5879) + 60 μ*M* indomethacin (Sigma-Aldrich I7378)), induction medium + 0.5 μ*M* rosiglitazone (Sigma-Aldrich R2408), or maintained in normal preadipocyte media (control). After 3 d, initial induction media was changed to insulin-containing media (DMEM/F12 + 10% FBS + 17 n*M* insulin), then was refreshed every 2d. Adipocyte differentiation was assessed on a ThermoScientific ArrayScan XTI automated microscope at 0, 3, and 7 d post-induction. Adipocyte proliferation was assessed in 96-well plates using an MTS assay (Promega, Madison, WI, USA) according to the manufacturer’s instructions. Proliferation was examined colourimetrically at time of induction initiation (2 d post-plating), and at 3 d post-induction (without induction medium [-ind.; control] or with induction medium [+ind.]). Adipocyte RNA was isolated from separate cells at 5d post-induction. Cells were treated with control medium, induction medium, or induction medium+rosiglitazone for 5d, then RNA was collected and isolated. Cells were differentiated with control medium, induction medium, or induction medium+rosiglitazone for 5d, then RNA was collected and isolated.

### mRNA processing and PCR

mRNA was performed using a standard TRIzol extraction (Life Technologies 15596). Tissue was homogenized in 500 μl lysis buffer. Reverse-transcriptase (RT-) PCR was completed using SuperScript II Reverse Transcriptase (Life Technologies) and quantitative (q-) PCR was completed using Taqman or SYBR Green. Several Life Technologies primers were used: Cd11b (F: GGATCATAGGCGCCCACTT, R: TCCTTACCCCCACTCAGAGACT); Ccl2 (F: AACCTGGATCGGAACCAAATG, R: AAGTGCTTGAGGTGGTTGTGG); AdipoQ: (F: CCCAGTCATGCCGAAGATGA, R: CACAAGTTCCCTTGGGTGGA); Tnf: Mm00443258_m1; IL-1b: Mm00434228_m1; IL-10: Mm00439614_m1; Creb1: Mm00501607_m1; Pnpla2: Mm00503040_m1; Fabp4: Mm00445878_m1; Cebpb: Mm00843434_s1; Pparg: Mm01184322_m1; Cidea: Mm00432554_m1; Ucp1: Mm01244861_m1; and Rheb: Mm00474045_m1. All genes were run in triplicate and normalized to 18S (SYBR – F: TTCGGAACTGAGGCCATGAT, R: TTTCGCTCTGGTCCGTCTTG; Taqman – Life Technologies 4319413E) for each individual sample. miR-155 deletion was confirmed in KO WAT: miR-155-5p was detectable in WT WAT samples from female mice (Supplemental Fig. S1g). The slight detection from KO samples (1% of WT levels) may have been from background amplification.

### Image analysis

For analysis, all researchers were blind with respect to treatment group. Images for analysis were collected on a Zeiss Axioplan 2 microscope, and image analysis was performed on MetaMorph. For adipocyte diameter analysis, three images per animal were collected. We manually circled adipocytes (>150 cells per animal) and measured adipocyte diameter. For lipid-containing cell density, 4+ images from each well (1 well per animal) were analyzed at 0, 3, and 7 d post-induction.

### Statistics

Immunohistological, morphometric, and gene expression levels were analyzed using one- or two-way ANOVA, followed by Tukey, Dunn’s, or Holm-Sidak *post-hoc* tests. SigmaPlot 12.0 (Systat, San Jose, CA, USA) and InStat 3 (GraphPad, La Jolla, CA, USA) were used to analyze the data. Mean differences were considered significant when p < 0.05. All data are plotted as mean ± SEM.

## Additional Information

**How to cite this article**: Gaudet, A. D. *et al*. miR-155 Deletion in Female Mice Prevents Diet-Induced Obesity. *Sci. Rep.* **6**, 22862; doi: 10.1038/srep22862 (2016).

## Supplementary Material

Supplementary Information

## References

[b1] Ogden C. L. . “Prevalence of childhood and adult obesity in the United States, 2011–2012”. JAMA 311, 806–814 (2014). 24570244 10.1001/jama.2014.732 PMC4770258

[b2] Bastien M. . “Overview of epidemiology and contribution of obesity to cardiovascular disease”. Prog Cardiovasc Dis 56, 369–381 (2014). 24438728 10.1016/j.pcad.2013.10.016

[b3] Gray S. L. & Vidal-Puig A. J. “Adipose tissue expandability in the maintenance of metabolic homeostasis”. Nutr Rev 65, S7–12 (2007). 17605308 10.1111/j.1753-4887.2007.tb00331.x

[b4] King N. A. . “Metabolic and behavioral compensatory responses to exercise interventions: barriers to weight loss”. Obesity (Silver Spring) 15, 1373–1383 (2007). 17557973 10.1038/oby.2007.164

[b5] Nicklas J. M. . “Successful weight loss among obese U.S. adults”. Am J Prev Med 42, 481–485 (2012). 22516488 10.1016/j.amepre.2012.01.005 PMC3339766

[b6] Krol J., Loedige I. & Filipowicz W. “The widespread regulation of microRNA biogenesis, function and decay”. Nat Rev Genet 11, 597–610 (2010). 20661255 10.1038/nrg2843

[b7] Friedman R. C. . “Most mammalian mRNAs are conserved targets of microRNAs”. Genome Res 19, 92–105 (2009). 18955434 10.1101/gr.082701.108 PMC2612969

[b8] Liu W. . “miR-133a regulates adipocyte browning *in vivo*”. PLoS Genet 9, e1003626 (2013). 23874225 10.1371/journal.pgen.1003626 PMC3708806

[b9] Seale P. . “Prdm16 determines the thermogenic program of subcutaneous white adipose tissue in mice”. J Clin Invest 121, 96–105 (2011). 21123942 10.1172/JCI44271 PMC3007155

[b10] Ohno H. . “PPARgamma agonists induce a white-to-brown fat conversion through stabilization of PRDM16 protein”. Cell Metab 15, 395–404 (2012). 22405074 10.1016/j.cmet.2012.01.019 PMC3410936

[b11] Frontini A. & Cinti S. “Distribution and development of brown adipocytes in the murine and human adipose organ”. Cell Metab 11, 253–256 (2010). 20374956 10.1016/j.cmet.2010.03.004

[b12] Leonardsson G. . “Nuclear receptor corepressor RIP140 regulates fat accumulation”. Proc Natl Acad Sci USA 101, 8437–8442 (2004). 15155905 10.1073/pnas.0401013101 PMC420412

[b13] Cederberg A. . “FOXC2 is a winged helix gene that counteracts obesity, hypertriglyceridemia, and diet-induced insulin resistance”. Cell 106, 563–573 (2001). 11551504 10.1016/s0092-8674(01)00474-3

[b14] Liu S., Yang Y. & Wu J. “TNFalpha-induced up-regulation of miR-155 inhibits adipogenesis by down-regulating early adipogenic transcription factors”. Biochem Biophys Res Commun 414, 618–624 (2011). 21986534 10.1016/j.bbrc.2011.09.131

[b15] Hou L. . “Critical role of miR-155/FoxO1/ROS axis in the regulation of non-small cell lung carcinomas”. Tumour Biol (2015). 10.1007/s13277-015-4335-9 26548866

[b16] Chakrabarti P. . “SIRT1 controls lipolysis in adipocytes via FOXO1-mediated expression of ATGL”. J Lipid Res 52, 1693–1701 (2011). 21743036 10.1194/jlr.M014647 PMC3151689

[b17] Kim J. Y. . “The adipose tissue triglyceride lipase ATGL/PNPLA2 is downregulated by insulin and TNF-alpha in 3T3-L1 adipocytes and is a target for transactivation by PPARgamma”. Am J Physiol Endocrinol Metab 291, E115–E127 (2006). 16705060 10.1152/ajpendo.00317.2005

[b18] Hotamisligil G. S. “Inflammation and metabolic disorders”. Nature 444, 860–867 (2006). 17167474 10.1038/nature05485

[b19] Lumeng C. N., Bodzin J. L. & Saltiel A. R. “Obesity induces a phenotypic switch in adipose tissue macrophage polarization”. J Clin Invest 117, 175–184 (2007). 17200717 10.1172/JCI29881 PMC1716210

[b20] Cai X. . “Re-polarization of tumor-associated macrophages to pro-inflammatory M1 macrophages by microRNA-155”. J Mol Cell Biol 4, 341–343 (2012). 22831835 10.1093/jmcb/mjs044

[b21] Rogers P. & Webb G. P. “Estimation of body fat in normal and obese mice”. Br J Nutr 43, 83–86 (1980). 7370219 10.1079/bjn19800066

[b22] Hatori M. . “Time-restricted feeding without reducing caloric intake prevents metabolic diseases in mice fed a high-fat diet”. Cell Metab 15, 848–860 (2012). 22608008 10.1016/j.cmet.2012.04.019 PMC3491655

[b23] Demas G. E. “The energetics of immunity: a neuroendocrine link between energy balance and immune function”. Horm Behav 45, 173–180 (2004). 15047012 10.1016/j.yhbeh.2003.11.002

[b24] Klaus S. “Adipose tissue as a regulator of energy balance”. Curr Drug Targets 5, 241–250 (2004). 15058310 10.2174/1389450043490523

[b25] Chen Y. . “miR-155 regulates differentiation of brown and beige adipocytes via a bistable circuit”. Nat Commun 4, 1769 (2013). 23612310 10.1038/ncomms2742 PMC3644088

[b26] Nazari-Jahantigh M. . “MicroRNA-155 promotes atherosclerosis by repressing Bcl6 in macrophages”. J Clin Invest 122, 4190–4202 (2012). 23041630 10.1172/JCI61716 PMC3484435

[b27] Hansen J. B. & Kristiansen K. “Regulatory circuits controlling white versus brown adipocyte differentiation”. Biochem J 398, 153–168 (2006). 16898874 10.1042/BJ20060402 PMC1550312

[b28] Aune U. L., Ruiz L. & Kajimura S. “Isolation and differentiation of stromal vascular cells to beige/brite cells”. J Vis Exp (2013). 10.3791/50191 PMC3641667 23568137

[b29] Kajimura S. . “Initiation of myoblast to brown fat switch by a PRDM16-C/EBP-beta transcriptional complex”. Nature 460, 1154–1158 (2009). 19641492 10.1038/nature08262 PMC2754867

[b30] Ahmadian M. . “Adipose overexpression of desnutrin promotes fatty acid use and attenuates diet-induced obesity”. Diabetes 58, 855–866 (2009). 19136649 10.2337/db08-1644 PMC2661591

[b31] Bauche I. B. . “Overexpression of adiponectin targeted to adipose tissue in transgenic mice: impaired adipocyte differentiation”. Endocrinology 148, 1539–1549 (2007). 17204560 10.1210/en.2006-0838

[b32] Hotamisligil G. S. . “Uncoupling of obesity from insulin resistance through a targeted mutation in aP2, the adipocyte fatty acid binding protein”. Science 274, 1377–1379 (1996). 8910278 10.1126/science.274.5291.1377

[b33] Lu M. . “Brain PPAR-gamma promotes obesity and is required for the insulin-sensitizing effect of thiazolidinediones”. Nat Med 17, 618–622 (2011). 21532596 10.1038/nm.2332 PMC3380629

[b34] Jones J. R. . “Deletion of PPARgamma in adipose tissues of mice protects against high fat diet-induced obesity and insulin resistance”. Proc Natl Acad Sci USA 102, 6207–6212 (2005). 15833818 10.1073/pnas.0306743102 PMC556131

[b35] Ravinet T. C. . “CB1 cannabinoid receptor knockout in mice leads to leanness, resistance to diet-induced obesity and enhanced leptin sensitivity”. Int J Obes Relat Metab Disord 28, 640–648 (2004). 14770190 10.1038/sj.ijo.0802583

[b36] Reuter T. Y. “Diet-induced models for obesity and type 2 diabetes,” 4, 3–8 (2007).

[b37] Klein S. L. “Immune cells have sex and so should journal articles”. Endocrinology 153, 2544–2550 (2012). 22434079 10.1210/en.2011-2120 PMC3359602

[b38] Beery A. K. & Zucker I. “Sex bias in neuroscience and biomedical research”. Neurosci Biobehav Rev 35, 565–572 (2011). 20620164 10.1016/j.neubiorev.2010.07.002 PMC3008499

[b39] Clayton J. A. & Collins F. S. “Policy: NIH to balance sex in cell and animal studies”. Nature 509, 282–283 (2014). 24834516 10.1038/509282a PMC5101948

[b40] Yasmeen R. . “Autocrine function of aldehyde dehydrogenase 1 as a determinant of diet- and sex-specific differences in visceral adiposity”. Diabetes 62, 124–136 (2013). 22933113 10.2337/db11-1779 PMC3526050

[b41] Schneider J. E. “Metabolic and hormonal control of the desire for food and sex: implications for obesity and eating disorders”. Horm Behav 50, 562–571 (2006). 16875692 10.1016/j.yhbeh.2006.06.023

[b42] Butovsky O. . “Targeting miR-155 restores abnormal microglia and attenuates disease in SOD1 mice”. Ann Neurol 77, 75–99 (2015). 25381879 10.1002/ana.24304 PMC4432483

[b43] Dai R. . “Sex differences in the expression of lupus-associated miRNAs in splenocytes from lupus-prone NZB/WF1 mice”. Biol Sex Differ 4, 19 (2013). 24175965 10.1186/2042-6410-4-19 PMC3843556

[b44] Fonken L. K. . “MicroRNA-155 deletion reduces anxiety- and depressive-like behaviors in mice”. Psychoneuroendocrinology 63, 362–369 (2016). 26555429 10.1016/j.psyneuen.2015.10.019 PMC13014412

[b45] Kelly M. A. . “Locomotor activity in D2 dopamine receptor-deficient mice is determined by gene dosage, genetic background, and developmental adaptations”. J Neurosci 18, 3470–3479 (1998). 9547254 10.1523/JNEUROSCI.18-09-03470.1998 PMC6792649

[b46] Keeney B. K. . “Differential response to a selective cannabinoid receptor antagonist (SR141716: rimonabant) in female mice from lines selectively bred for high voluntary wheel-running behaviour”. Behav Pharmacol 19, 812–820 (2008). 19020416 10.1097/FBP.0b013e32831c3b6b

[b47] Lightfoot J. T. “Sex hormones’ regulation of rodent physical activity: a review”. Int J Biol Sci 4, 126–132 (2008). 18449357 10.7150/ijbs.4.126 PMC2359866

[b48] Morgan M. A., Schulkin J. & Pfaff D. W. “Estrogens and non-reproductive behaviors related to activity and fear”. Neurosci Biobehav Rev 28, 55–63 (2004). 15036933 10.1016/j.neubiorev.2003.11.017

[b49] Manchado C. . “CCAAT/enhancer-binding proteins alpha and beta in brown adipose tissue: evidence for a tissue-specific pattern of expression during development”. Biochem J 302 (Pt 3), 695–700 (1994). 7945193 10.1042/bj3020695 PMC1137287

[b50] Tanaka T. . “Defective adipocyte differentiation in mice lacking the C/EBPbeta and/or C/EBPdelta gene”. EMBO J 16, 7432–7443 (1997). 9405372 10.1093/emboj/16.24.7432 PMC1170343

[b51] Jimenez-Preitner M. . “Plac8 is an inducer of C/EBPbeta required for brown fat differentiation, thermoregulation, and control of body weight”. Cell Metab 14, 658–670 (2011). 21982742 10.1016/j.cmet.2011.08.008

[b52] Nedergaard J. . “PPARgamma in the control of brown adipocyte differentiation”. Biochim Biophys Acta 1740, 293–304 (2005). 15949696 10.1016/j.bbadis.2005.02.003

[b53] Kershaw E. E. . “PPARgamma regulates adipose triglyceride lipase in adipocytes *in vitro* and *in vivo*”. Am J Physiol Endocrinol Metab 293, E1736–E1745 (2007). 17848638 10.1152/ajpendo.00122.2007 PMC2819189

[b54] Huijsman E. . “Adipose triacylglycerol lipase deletion alters whole body energy metabolism and impairs exercise performance in mice”. Am J Physiol Endocrinol Metab 297, E505–E513 (2009). 19491295 10.1152/ajpendo.00190.2009

[b55] Mottillo E. P. . “Coupling of lipolysis and *de novo* lipogenesis in brown, beige, and white adipose tissues during chronic beta3-adrenergic receptor activation”. J Lipid Res 55, 2276–2286 (2014). 25193997 10.1194/jlr.M050005 PMC4617130

[b56] Li Y. . “Taking control over intracellular fatty acid levels is essential for the analysis of thermogenic function in cultured primary brown and brite/beige adipocytes”. EMBO Rep 15, 1069–1076 (2014). 25135951 10.15252/embr.201438775 PMC4253847

[b57] Klaus S. . “The uncoupling protein UCP: a membraneous mitochondrial ion carrier exclusively expressed in brown adipose tissue”. Int J Biochem 23, 791–801 (1991). 1773883 10.1016/0020-711x(91)90062-r

[b58] Divakaruni A. S. & Brand M. D. “The regulation and physiology of mitochondrial proton leak”. Physiology (Bethesda) 26, 192–205 (2011). 21670165 10.1152/physiol.00046.2010

[b59] Feldmann H. M. . “UCP1 ablation induces obesity and abolishes diet-induced thermogenesis in mice exempt from thermal stress by living at thermoneutrality”. Cell Metab 9, 203–209 (2009). 19187776 10.1016/j.cmet.2008.12.014

[b60] Yang R. . “RNAi-mediated germline knockdown of FABP4 increases body weight but does not improve the deranged nutrient metabolism of diet-induced obese mice”. Int J Obes (Lond) 35, 217–225 (2011). 20603627 10.1038/ijo.2010.128 PMC3056343

[b61] Kralisch S. & Fasshauer M. “Adipocyte fatty acid binding protein: a novel adipokine involved in the pathogenesis of metabolic and vascular disease?” Diabetologia 56, 10–21 (2013). 23052058 10.1007/s00125-012-2737-4

[b62] Huang C. . “Arg(9)(7)(2) insulin receptor substrate-1 inhibits endothelial nitric oxide synthase expression in human endothelial cells by upregulating microRNA-155”. Int J Mol Med 36, 239–248 (2015). 25902041 10.3892/ijmm.2015.2192

[b63] Tseng Y. H. . “Prediction of preadipocyte differentiation by gene expression reveals role of insulin receptor substrates and necdin”. Nat Cell Biol 7, 601–611 (2005). 15895078 10.1038/ncb1259

[b64] Araki E. . “Alternative pathway of insulin signalling in mice with targeted disruption of the IRS-1 gene”. Nature 372, 186–190 (1994). 7526222 10.1038/372186a0

[b65] Tamemoto H. . “Insulin resistance and growth retardation in mice lacking insulin receptor substrate-1”. Nature 372, 182–186 (1994). 7969452 10.1038/372182a0

[b66] Hotamisligil G. S., Shargill N. S. & Spiegelman B. M. “Adipose expression of tumor necrosis factor-alpha: direct role in obesity-linked insulin resistance”. Science 259, 87–91 (1993). 7678183 10.1126/science.7678183

[b67] Uysal K. T. . “Protection from obesity-induced insulin resistance in mice lacking TNF-alpha function”. Nature 389, 610–614 (1997). 9335502 10.1038/39335

[b68] Marshall S. “Role of insulin, adipocyte hormones, and nutrient-sensing pathways in regulating fuel metabolism and energy homeostasis: a nutritional perspective of diabetes, obesity, and cancer”. Sci STKE 2006, re7 (2006). 16885148 10.1126/stke.3462006re7

[b69] Chakrabarti P. . “The mammalian target of rapamycin complex 1 regulates leptin biosynthesis in adipocytes at the level of translation: the role of the 5′-untranslated region in the expression of leptin messenger ribonucleic acid”. Mol Endocrinol 22, 2260–2267 (2008). 18653778 10.1210/me.2008-0148 PMC2582535

[b70] Maeda N. . “Diet-induced insulin resistance in mice lacking adiponectin/ACRP30”. Nat Med 8, 731–737 (2002). 12068289 10.1038/nm724

[b71] Szewczyk-Golec K., Wozniak A. & Reiter R. J. “Inter-relationships of the chronobiotic, melatonin, with leptin and adiponectin: implications for obesity”. J Pineal Res 59, 277–291 (2015). 26103557 10.1111/jpi.12257

[b72] Barnea M. . “The circadian clock machinery controls adiponectin expression”. Mol Cell Endocrinol 399, 284–287 (2015). 25448847 10.1016/j.mce.2014.10.018

[b73] Maeda N. . “PPARgamma ligands increase expression and plasma concentrations of adiponectin, an adipose-derived protein”. Diabetes 50, 2094–2099 (2001). 11522676 10.2337/diabetes.50.9.2094

[b74] Fasshauer M. . “Hormonal regulation of adiponectin gene expression in 3T3-L1 adipocytes”. Biochem Biophys Res Commun 290, 1084–1089 (2002). 11798186 10.1006/bbrc.2001.6307

[b75] Schmid B. . “Inhibition of fatty acid synthase prevents preadipocyte differentiation”. Biochem Biophys Res Commun 328, 1073–1082 (2005). 15707987 10.1016/j.bbrc.2005.01.067

[b76] Moon H. S. . “Inhibitory effect of (-)-epigallocatechin-3-gallate on lipid accumulation of 3T3-L1 cells”. Obesity (Silver Spring) 15, 2571–2582 (2007). 18070748 10.1038/oby.2007.309

[b77] Stenbit A. E. . “GLUT4 heterozygous knockout mice develop muscle insulin resistance and diabetes”. Nat Med 3, 1096–1101 (1997). 9334720 10.1038/nm1097-1096

[b78] Shepherd P. R. . “Adipose cell hyperplasia and enhanced glucose disposal in transgenic mice overexpressing GLUT4 selectively in adipose tissue”. J Biol Chem 268, 22243–22246 (1993). 8226728

[b79] Abel E. D. . “Adipose-selective targeting of the GLUT4 gene impairs insulin action in muscle and liver”. Nature 409, 729–733 (2001). 11217863 10.1038/35055575

[b80] Yudkin J. S. . “C-reactive protein in healthy subjects: associations with obesity, insulin resistance, and endothelial dysfunction: a potential role for cytokines originating from adipose tissue?” Arterioscler Thromb Vasc Biol 19, 972–978 (1999). 10195925 10.1161/01.atv.19.4.972

[b81] Wisse B. E. “The inflammatory syndrome: the role of adipose tissue cytokines in metabolic disorders linked to obesity”. J Am Soc Nephrol 15, 2792–2800 (2004). 15504932 10.1097/01.ASN.0000141966.69934.21

[b82] Makki K., Froguel P. & Wolowczuk I. “Adipose tissue in obesity-related inflammation and insulin resistance: cells, cytokines, and chemokines”. ISRN Inflamm 2013, 139239 (2013). 24455420 10.1155/2013/139239 PMC3881510

[b83] Lumeng C. N. . “Phenotypic switching of adipose tissue macrophages with obesity is generated by spatiotemporal differences in macrophage subtypes”. Diabetes 57, 3239–3246 (2008). 18829989 10.2337/db08-0872 PMC2584129

[b84] Martinez-Nunez R. T., Louafi F. & Sanchez-Elsner T. “The interleukin 13 (IL-13) pathway in human macrophages is modulated by microRNA-155 via direct targeting of interleukin 13 receptor alpha1 (IL13Ralpha1)”. J Biol Chem 286, 1786–1794 (2011). 21097505 10.1074/jbc.M110.169367 PMC3023473

[b85] Tili E. . “Modulation of miR-155 and miR-125b levels following lipopolysaccharide/TNF-alpha stimulation and their possible roles in regulating the response to endotoxin shock”. J Immunol 179, 5082–5089 (2007). 17911593 10.4049/jimmunol.179.8.5082

[b86] Worm J. . “Silencing of microRNA-155 in mice during acute inflammatory response leads to derepression of c/ebp Beta and down-regulation of G-CSF”. Nucleic Acids Res 37, 5784–5792 (2009). 19596814 10.1093/nar/gkp577 PMC2761263

[b87] Louafi F., Martinez-Nunez R. T. & Sanchez-Elsner T. “MicroRNA-155 targets SMAD2 and modulates the response of macrophages to transforming growth factor-{beta}”. J Biol Chem 285, 41328–41336 (2010). 21036908 10.1074/jbc.M110.146852 PMC3009858

[b88] Gunasekaran M. K. . “Inflammation triggers high mobility group box 1 (HMGB1) secretion in adipose tissue, a potential link to obesity”. Cytokine 64, 103–111 (2013). 23938155 10.1016/j.cyto.2013.07.017

[b89] Arrigo T. . “High-mobility group protein B1: a new biomarker of metabolic syndrome in obese children”. Eur J Endocrinol 168, 631–638 (2013). 23384711 10.1530/EJE-13-0037

[b90] Nativel B. . “Soluble HMGB1 is a novel adipokine stimulating IL-6 secretion through RAGE receptor in SW872 preadipocyte cell line: contribution to chronic inflammation in fat tissue”. PLoS One 8, e76039 (2013). 24073286 10.1371/journal.pone.0076039 PMC3779194

[b91] Tian F. J. . “Elevated microRNA-155 promotes foam cell formation by targeting HBP1 in atherogenesis”. Cardiovasc Res 103, 100–110 (2014). 24675724 10.1093/cvr/cvu070

[b92] Thai T. H. . “Regulation of the germinal center response by microRNA-155”. Science 316, 604–608 (2007). 17463289 10.1126/science.1141229

[b93] Donnelly D. J. . “Deficient CX3CR1 signaling promotes recovery after mouse spinal cord injury by limiting the recruitment and activation of Ly6Clo/iNOS+macrophages”. J Neurosci 31, 9910–9922 (2011). 21734283 10.1523/JNEUROSCI.2114-11.2011 PMC3139517

[b94] Fonken L. K. . “Light at night increases body mass by shifting the time of food intake”. Proc Natl Acad Sci USA 107, 18664–18669 (2010). 20937863 10.1073/pnas.1008734107 PMC2972983

[b95] Demas G. E. . “Metabolic costs of mounting an antigen-stimulated immune response in adult and aged C57BL/6J mice”. Am J Physiol 273, R1631–R1637 (1997). 9374803 10.1152/ajpregu.1997.273.5.R1631

[b96] Kaiyala K. J. & Schwartz M. W. “Toward a more complete (and less controversial) understanding of energy expenditure and its role in obesity pathogenesis”. Diabetes 60, 17–23 (2011). 21193735 10.2337/db10-0909 PMC3012169

[b97] Liu C. . “Central IKKbeta inhibition prevents air pollution mediated peripheral inflammation and exaggeration of type II diabetes”. Part Fibre Toxicol 11, 53 (2014). 25358444 10.1186/s12989-014-0053-5 PMC4226918

[b98] Hausman D. B., Park H. J. & Hausman G. J. “Isolation and culture of preadipocytes from rodent white adipose tissue”. Methods Mol Biol 456, 201–219 (2008). 18516563 10.1007/978-1-59745-245-8_15

