# Supplementary material for: miR-155 Deletion in Female Mice Prevents Diet-Induced Obesity
Source: Sci Rep. 2016 Mar 8;6:22862. doi: 10.1038/srep22862 (PMC4782173; doi:10.1038/srep22862)
Supplement: Supplementary Information [file srep22862-s1.pdf]

Supplementary Information File

microRNA-155 Deletion in Female Mice Prevents Diet-Induced Obesity

Andrew D. Gaudet*, Laura K. Fonken*, Liubov V. Gushchina, Taryn G. Aubrecht, Santosh K. Maurya, Muthu Periasamy, Randy J. Nelson, and Phillip G. Popovich

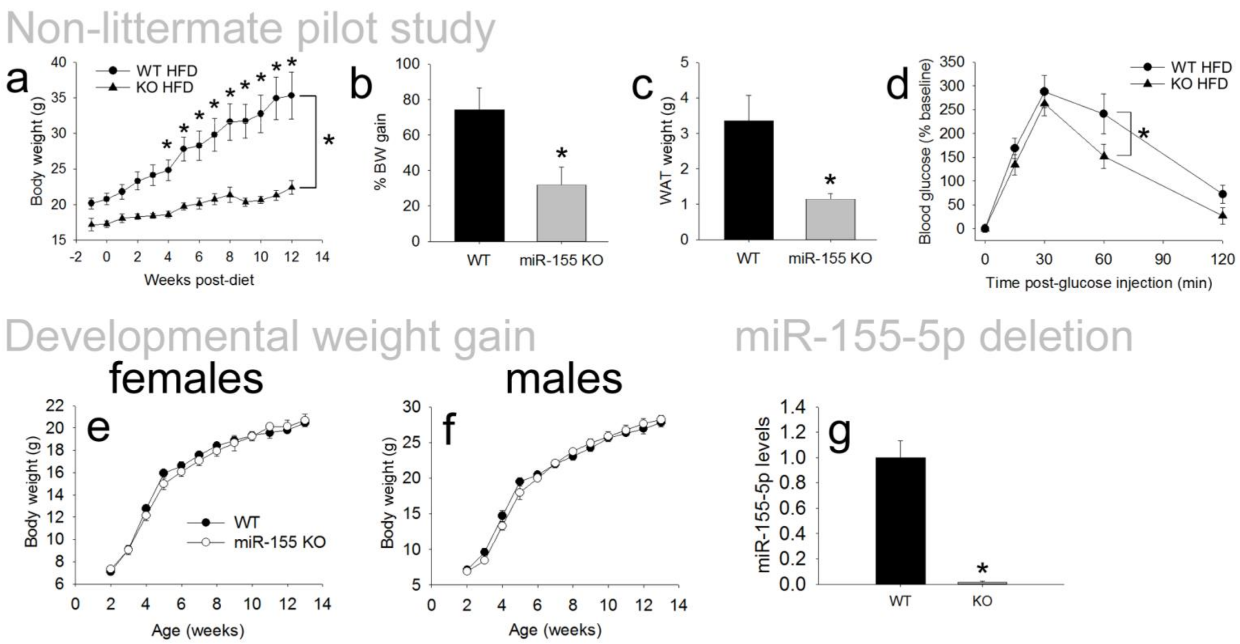

Supplemental Figure S1. A pilot experiment using female mice derived from WT or KO parents (not littermates) that led to studying littermates from heterozygote parents using CD and HFD (see Fig. 1); and developmental weight gain in littermate WT and KO mice. After noting that miR-155 KO mice from KO parents weighed less than WT mice, we placed mice on HFD. (a) miR-155 KO mice gained significantly less BW on HFD than WT mice. miR-155 KO mice on HFD weighed significantly less than HFD WT mice from 4-12 weeks after HFD start. Overall, miR-155 KO HFD mice weighed significantly less than WT HFD mice. (b) miR-155 KO mice gained significantly less weight from the start of the experiment. (c) Gonadal WAT from miR-155 KO mice weighed significantly less than WT WAT. (d) miR-155 KO mice on HFD recovered from glucose challenge significantly faster than WT mice. (e,f) Developmental weight gain was similar in WT and miR-155 KO mice (females, e; males, f) from heterozygote parents. (g) miR-155-5p was not expressed in WAT from female KO mice. * indicates $p < 0.05$.

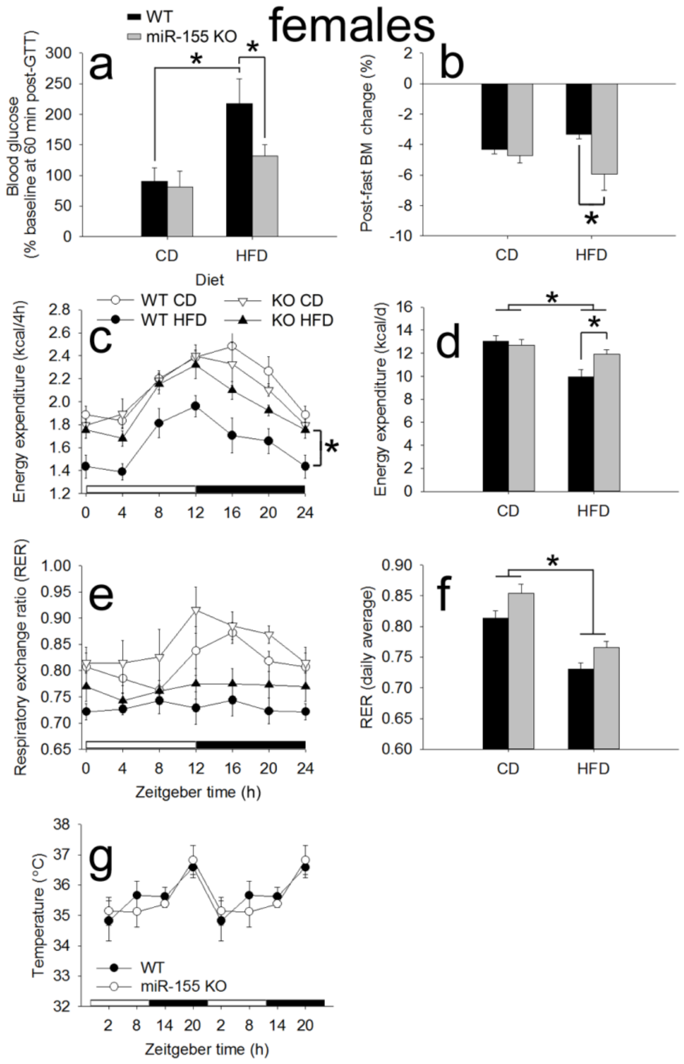

Supplemental Figure S2. Female miR-155 KO mice display altered energetics and metabolism (see also Fig. 3). In Fig. 3e-h, only HFD data are presented for clarity. Here, CD and HFD data are presented together. (a) Compared to WT mice, miR-155 KO mice fed HFD improved glucose clearance. At 60 min post-glucose challenge, miR-155 KO mice fed HFD improved glucose clearance by 39%. (b) After overnight fast, miR-155 KO mice had exaggerated BW loss compared to WT mice (HFD only). (c,d) Compared to WT mice, miR-155 KO mice fed HFD increased energy expenditure. (e,f) Respiratory exchange ratio was reduced in mice fed HFD; however, there were no significant differences in respiratory exchange ratio between genotypes. (g) Female miR-155 KO mice did not have significantly altered rectal temperature throughout the day.

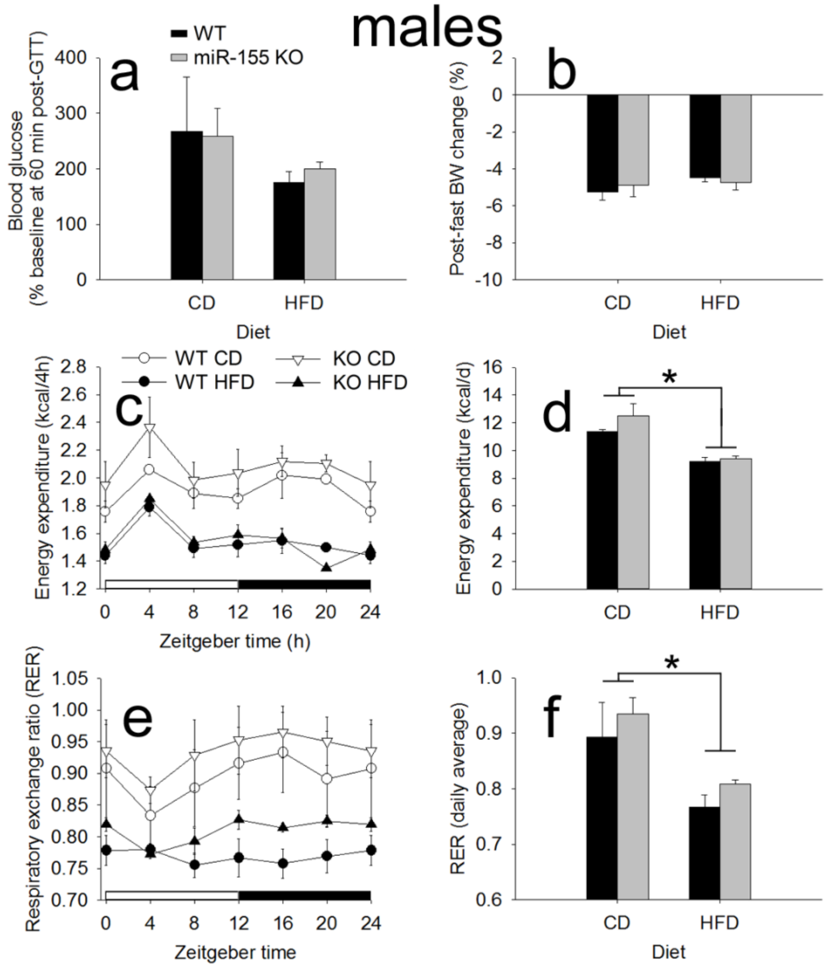

Supplemental Figure S3. Male miR-155 KO mice do not show altered energetics and metabolism (see also Fig. 3). In Fig. 3m-p, only HFD data are presented for clarity. Here, CD and HFD data are presented together. (a) WT and miR-155 KO mice had similar glucose clearance. (b) After overnight fast, WT and miR-155 KO mice had similar BW loss. (c,d) Energy expenditure was not significantly different between genotypes. (e,f) Respiratory exchange ratio was reduced in mice fed HFD; however, there were no significant differences in respiratory exchange ratio between genotypes.

miR-155-5p validated & predicted targets: A partial list

genes in red = validated targets

SOURCES:
https://cm.jefferson.edu/rna22v1.0-mus_musculus/InputController?identifier=mmu-miR-155
http://www.targetscan.org/cgi-bin/targetscan/vert_61/targetscan.cgi?mirg=mmu-miR-155
http://www.microrna.org/microrna/getTargets.do?matureName=mmu-miR-155&startIndex=350&organism=10090
http://www.mirbase.org/cgi-bin/mirna_entry.pl?acc=MI0000177

**ADIPOGENESIS**			**MACROPHAGES**			
**Transcriptional regulators**	**C/EBPb**		**TFs controling macrophage phenotype**	**C/EBPb**		
	**Creb1**			**Spi1**	gene for PU.1 transcription factor	
				**Creb1**		
**JAK/STAT pathway**	**Socs1**			**Bcl6**		
	Jak1			**Nr1h3**	LXRalpha gene	
	Jak2			Ppargc1 b	PPAR, gamma, coactivator 1 beta gene (PGC1beta)	
	Stat3					
	Stat5a		**TNF signaling**	**Tnfa**	miR-155 increases TNFa	
				Ikbkg	inhibitor of kappaB kinase gamma gene	
**Insulin signaling**	**Foxo1**			Traf3	TNF receptor-associated factor 3 gene	
	insulin receptor					
	insulin II		**TGF signaling**	TGFbeta receptor 2		
	Insig1			Tgf alpha		
	Insrr			Tgfbr1	TGF, beta receptor 1 gene	
	Igfbp1					
			**Other, pro-inflammatory**	IL-12 receptor beta 1		
**Leptin signaling**	Lepr			MMP-12	various MMPs, ADAMs, ADAM-TS	
	Leprot			Ccr7		
**Steroid-related**	Esr1		**Other, anti-inflammatory**	**Il-13Ral**		
	Esrra			Msr1	Macrophage scavenger receptor 1 (Msr1)	
	Pgr			Cd200r3	CD200 receptor 3 gene	
	Pgrmc1			CD200		
	Gmeb1			IL-4Ra		
	Trip13			Mrc1, 2	mannose receptor, C type 1 gene (and 2)	
				Adpor1	adiponectin receptor 1 gene	
**Other**	**Tnfa**	miR-155 increases TNFa				
	**Rheb**		**Other, potentially important**	**Rheb**		
	lipoprotein lipase			**HMGB1**		
	Lrp8	LDL receptor-related protein 8		**SHIP1**		
	Adiponectin receptor			**Socs1**		
	Mc5r	melanocortin 5 receptor gene		FGF-6		
	Apob	apolipoprotein B gene		P2rx7		From: Gaudet et al.
	Lpin1	lipin 1 gene		P2rx4		*microRNA-155 Deletion in Female Mice Prevents Diet-Induced Obesity*

Supplemental Table S1. miR-155-5p validated (red) and predicted mRNA targets. Analysis of potential targets were performed using Targetscan.org, microrna.org, mirbase.org, and the microRNA resource from Thomas Jefferson University. Validated targets were identified in published studies (see text for citations).
